# Different functions for the domains of the *Arabidopsis thaliana* RMI1 protein in DNA cross-link repair, somatic and meiotic recombination

**DOI:** 10.1093/nar/gkt730

**Published:** 2013-08-16

**Authors:** Simone Bonnet, Alexander Knoll, Frank Hartung, Holger Puchta

**Affiliations:** Karlsruhe Institute of Technology, Botanical Institute II, Hertzstrasse 16, 76187 Karlsruhe, Germany

## Abstract

Recombination intermediates, such as double Holliday junctions, can be resolved by nucleases or dissolved by the combined action of a DNA helicase and a topoisomerase. In eukaryotes, dissolution is mediated by the RTR complex consisting of a RecQ helicase, a type IA topoisomerase and the structural protein RecQ-mediated genome instability 1 (RMI1). Throughout eukaryotes, the RTR complex is involved in DNA repair and in the suppression of homologous recombination (HR) in somatic cells. Surprisingly, *Arabidopsis thaliana* mutants of topoisomerase 3α and *RMI1* are also sterile due to extensive chromosome breakage in meiosis I, indicating that both proteins are essential for meiotic recombination in plants. AtRMI1 harbours an N-terminal DUF1767 domain and two oligosaccharide binding (OB)-fold domains. To define specific roles for these individual domains, we performed complementation experiments on *Atrmi1* mutants with an *AtRMI1* full-length open reading frame (ORF) or deletion constructs lacking specific domains. We show that the DUF1767 domain and the OB-fold domain 1 are both essential for the function of AtRMI1 in DNA cross-link repair as well as meiotic recombination, but partially dispensable for somatic HR suppression. The OB-fold domain 2 is not necessary for either somatic or meiotic HR, but it seems to have a minor function in DNA cross-link repair.

## INTRODUCTION

The resolution of recombination intermediates, such as double Holliday junctions (dHJ), by endonucleases is an indispensable step for the proper segregation of homologous chromosomes in meiosis and to ensure genomic stability in somatic cells. The dissolution mechanism by the RTR complex is an alternative way to process recombination intermediates, such as dHJs (1,2). This mechanism was postulated first by Thaler and Stahl in 1988 (3) and requires a RecQ family DNA helicase and a type I topoisomerase.

RecQ helicases can be found in almost all pro- and eukaryotes (4). In most cases, loss of RecQ genes results in a hyper-recombination phenotype. Mutations in the *BLM*, *WRN* and *RECQ4* genes are the cause of severe hereditary diseases, namely, Bloom (5,6), Werner (7) and Rothmund–Thomson syndromes (8), respectively. Topoisomerases are sorted into two basic types that differ in their ability to create either single strand (type I) or double-strand breaks (type II). Each type can be subdivided into two families each, which have been defined either by their chemical properties (IA and IB) or by structural differences between the enzymes (IIA and IIB). There are three topoisomerases in yeast: TOP1, 2 and 3. In contrast to TOP1 and 2, which are well-characterized and involved in DNA replication (TOP1, type IB) or decatenation of linked chromosomes (TOP2, type IIA) (9), the main function of yeast TOP3 is in the dissolution reaction of DNA double-strand break repair by homologous recombination (HR) (10–12).

During the dissolution reaction, the two junctions of the dHJ are migrated towards each other by the adenosine triphosphate-driven activity of the DNA helicase. The generated hemicatenane structure is then processed by a type IA topoisomerase, which mediates the strand passage to untangle the two DNA double strands, resulting exclusively in non-crossover products. Specific RecQ helicases (Sgs1 in yeast and BLM in mammals) as well as type IA topoisomerases (topoisomerase 3 in yeast and 3α in mammals) were identified as proteins involved in this pathway (11,13,14).

Interestingly, a third protein named RecQ-mediated genome instability 1 (RMI1) (also called BLAP75) was found to be required for the dissolution mechanism. These three proteins form the evolutionarily highly conserved RTR complex (2,15–17).

The structural protein RMI1 possesses no catalytic function itself. Nevertheless, it is required to stimulate the formation of the RTR complex as well as the DNA-binding activity of the topoisomerase, and therefore the dissolution reaction, *in vitro* (18–20). Both the functions and the components of the RTR complex are conserved in eukaryotes (2,15–17). In mammals, RMI2 participates as a fourth complex partner with a stabilizing and dissolution stimulating function (21,22). In the model plant *Arabidopsis thaliana* (*A. thaliana*, abbreviated At in front of gene and protein names), the RTR complex is composed of the RecQ helicase RECQ4A, the type IA topoisomerase TOP3A and RMI1 (23). It has been shown that all three partners are required for the suppression of HR in somatic cells as well as in DNA cross-link repair (23,24). In addition to the somatic function, AtRMI1 and AtTOP3A also play an important role in meiotic recombination (23,25). Intriguingly, the *Atrecq4A* mutant is not sterile but has only minor meiotic defects (26,27). Thus, AtRMI1 and AtTOP3A have an essential role in meiosis independent of AtRECQ4A.

The crucial role of AtRMI1 in plant meiosis was surprising because a similar phenotype has not been reported in any other eukaryote. To define the meiotic function in comparison with the well-known functions of RMI1 homologues of other eukaryotes in DNA repair and the suppression of somatic HR, we investigated which parts of the *AtRMI1* gene are essential for mitotic or meiotic functions by mutating individual domains. Similar to its mammalian homologue, AtRMI1 is composed of an N-terminal domain of unknown function 1767 (DUF1767; pfam08585), a first oligonucleotide/oligosaccharide binding-fold (OB-fold) domain (OB1) followed by a second OB-fold domain (OB2) in the C-terminal part of the protein. The overall sequence identity between the RMI1 homologue in Arabidopsis and humans is low, but the domains are highly conserved. The function of the DUF1767 domain is still unclear, but it is thought to be important for the proper folding of the protein (28,29). In yeast and mammals, the OB-fold domain 1 in the N-terminal part of RMI1 mediates the interaction with the RecQ helicase and the type IA topoisomerase and is essential for the dHJ dissolution reaction (17,29,30). In humans, an interaction of RMI2 with the OB-fold domain 2 located in the C-terminal part of the protein has been described (29,31). This does not apply to yeast, as the RMI1 homologue is shorter than the mammalian homologue and possesses no OB2 fold domain. Additionally, no RMI2 homologue is present in the yeast genome (21,22). Interestingly, a homologue of RMI2 is present in plants (22), but no information about its function has been reported so far.

To define the function of the conserved AtRMI1 domains in DNA repair as well as in somatic and meiotic HR, we performed complementation experiments in *Atrmi1* mutants using the full-length ORF and several recombinant *AtRMI1* ORFs in which specific domains were deleted. Our results demonstrate that both the DUF1767 domain and the OB-fold domain 1 of the N-terminal part of AtRMI1 are necessary for the function of AtRMI1 in DNA repair. These domains are partially dispensable for the function of AtRMI1 in somatic HR suppression but not for the dissolution of meiotic recombination intermediates. The OB-fold domain 2 is neither necessary for HR in somatic cells nor for meiotic recombination. Nevertheless, it seems to have a minor function in DNA repair. Thus, for all three processes addressed, the domain requirements differ, indicating unique roles for AtRMI1 in all three pathways.

## MATERIALS AND METHODS

### Plant material and growth conditions

For the complementation experiments, the mutant lines *Atrmi1-1* (SALK_093589) and *Atrmi1-2* (SALK_094387) of *A. thaliana* ecotype Col-0 were used. These mutant lines from the Salk collection (32) have been previously described (23,25). To measure somatic HR, the IC9 reporter construct was used (33). For propagation and to obtain anthers for the analysis of meiosis in pollen mother cells, the plants were grown in a greenhouse in soil at constant 22°C (16 h light/8 h dark). To determine the somatic genotoxin sensitivity and HR frequency, the plants were grown under axenic conditions. After stratification at 4°C overnight, the seeds were surface sterilized with 4% sodium hypochlorite and sown on agar plates containing germination medium (GM: 4,9 g/l Murashige & Skoog including vitamins and MES [2-(N-morpholino)ethanesulfonic acid], 10 g/l sucrose and 0.76 g/l agar (adjusted to pH 5.7 with KOH). The plantlets were cultivated in a CU-36L4 plant culture chamber (Percival Scientific, Inc., Perry, IA, USA) under tightly controlled conditions with 16 h of light at 22°C and 8 h of dark at 20°C.

### Primers used for PCR-based genotyping of the mutant lines

Two primer pairs were used to genotype each of the *Atrmi1* mutant lines. The deletion of the *Atrmi1-1* mutant line could be detected by a pair of primers located upstream and downstream of the deletion in *AtRMI1* (5′-AACCGGAAACCTCAGTATC-3′/5′-CATTGATTGAAGACTGAGAGTG-3′). The second PCR was performed with one primer upstream of the deletion and one primer located within the deletion (5′-ATGTGTGATTTTGGCTGAAC-3′/5′-CTAAACGAGTACATTGTCAG-3′). To detect the wild-type allele of the *Atrmi1-2* line, one pair of primers was located upstream and downstream of the insertion site (5′-TTCACCATAGCCGAGTTAC-3′/5′-AGAAGCTCATACGTAGACTG-3′). The second primer pair contained one primer binding to the *AtRMI1* locus and one T-DNA-specific primer (5′-TTCACCATAGCCGAGTTAC-3′/5′-TCGGAACCACCATCAAACAG-3′).

### Plasmid construction and plant transformation

The constructs used for plant transformations are based on the binary plasmid pPZP201 (34) and contain an additional phosphinothricin (PPT) resistance cassette under the control of the CaMV 35 S gene promoter and terminator proximal to the RB of the T-DNA for the selection of transformed plants. The coding sequences of *AtRMI1* or the *AtRMI1* deletion variants are based on the cDNA sequence of *AtRMI1* [AY735746; (23)]. In the first step, we cloned a construct with the full-length cDNA sequence by combining 1050 bp upstream of the start codon for the promoter and 5′ UTR (primer pair 5′- GTGCCAACCAGCCAAGATTG-3′/5′- TTCTTCGCCGGCGAAATTTAG -3′), the full ORF (primer pair 5′- ATGCGTAGACGGCGCCTG-3′/5′-TCAAGGGGACAGAACAACA-3′) and 400 bp downstream of the stop codon for the 3′ UTR and terminator (primer pair 5′-TGATCCAGTACTCAACTAAAAG-3′/5′-CGTGTCTTATTTGGTCGAGTC-3′) using the In-Fusion Cloning system (Clontech, Mountain View, CA, USA). Based on this wild-type clone, four constructs with *AtRMI1* deletion variants were established: RMI1ΔDUF, in which we deleted bases 301–582 relative to the start codon, corresponding to residues 101–194 (primer pairs 5′-GTGCCAACCAGCCAAGATTG-3′/5′-GATCGGCGATTCATAGTCATTG-3′ and 5′-AATGCTAATGCAGGGCTTAAG-3′/5′-CGTGTCTTATTTGGTCGAGTC-3′); RMI1ΔOB1, in which we deleted bases 700–774 relative to the start codon, corresponding to residues 234–258 (primer pairs 5′-GTGCCAACCAGCCAAGATTG-3′/5′-ACCAGCAGGAGCCAAGACTT-3′ and 5′-GGAGGGATGGTTGAAGAACTA-3′/5′-CGTGTCTTATTTGGTCGAGTC-3′); RMI1ΔOB2, in which we deleted bases 1452–1932 relative to the start codon, corresponding to the residues 485–644 (primer pairs 5′-GTGCCAACCAGCCAAGATTG-3′/5′-GGTTTCTCTGTATTTGTAGACAGC-3′ and 5′-TGATCCAGTACTCAACTAAAAG-3′/5′-CGTGTCTTATTTGGTCGAGTC-3′); and RMI1ΔDUFΔOB1, in which we deleted residues 101–194 and 234–258 using the same primers as mentioned previously.

Agrobacterium-mediated transformation of *Atrmi1-1*, *Atrmi1-2* and wild-type plants (all in a homozygous IC9 background) was performed via the floral dip method (35). After the selection of transformed plants in the T1 generation by PPT resistance, the T2 generation was checked for a mendelian 3:1 segregation to obtain lines in which the transgenic T-DNA was inserted at a single genomic locus. After propagation, the T3 generation was tested for homozygous single locus lines through PPT resistance.

### RNA extraction and quantitative PCR

Total RNA was extracted from 2-week-old plantlets using the RNeasy Plant Mini Kit (Qiagen GmbH, Hilden, Germany) according to the manufacturer’s instructions. Reverse transcription was conducted using the RevertAid First-strand cDNA Synthesis Kit (Fermentas, St. Leon-Rot, Germany) according to the manufacturer’s instructions. Expression analysis was performed by quantitative PCR (45 cycles of amplification: 10 s, 95°C; 20 s 57°C; 40 s 72°C; detection at the amplification step) with SYBR Green I Master Mix (Roche Diagnostics GmbH, Mannheim, Germany). The results were normalized using the constitutively expressed *Actin2* gene (At3g18780) (5′-CAGATGCCCAGAAGTCTTG-3′/5′-GTGCTGTGATTTCTTTGCTC-3′) as an internal standard (36). The primer pair (5′-TAGACGGCGCCTGCAAC-3′/5′-AATACCAAAGCTCTGAACAG-3′) was designed to amplify a diagnostic amplicon of 97 bp in the *Atrmi1-2* mutant or an amplicon of 200 bp in the *Atrmi1-1* mutant (5′-ATTCACCGAGCAGCATCCAC-3′/5′-TACACCGCCTGAATCTGAAC-3′). The gene expression calculations were performed with the LightCycler 480 SW 1.5 software (Roche Diagnostics GmbH). The relative quantification was done after the PCR efficiency calculation from standard curves of both the target and reference amplicons that were generated from serial dilutions of wild-type cDNA. To determine Cp calling, the second derivative maximum method was used.

### Sensitivity assays

Sensitivity assays were performed as previously described (24). After 1 week of growth on solid GM medium, 10 plantlets were transferred to each well of a six-well plate containing 5 ml of liquid GM for the untreated control or 5 ml of liquid GM supplemented with cisplatin or methylmethane sulfonate (MMS) for final concentrations of 10 µM (cisplatin) and 60 ppm (MMS, equivalent to 708 µM at 25°C), respectively. After another 13 days of incubation, the fresh weight of the plants was measured. Results were calculated as fresh weight of treated plantlets relative to untreated plantlets of the same line.

### HR assays

The HR assays using the IC9 reporter construct were performed as described (24,33) by transferring 40 1-week-old plantlets from solid GM to both chambers of halved Petri dishes containing 10 ml of liquid GM each. To measure the recombination rate after the induction of DNA damage, the plantlets were treated with cisplatin at a final concentration of 3 µM. The plantlets were incubated for a total of 7 days in liquid medium, followed by a β-glucuronidase (GUS) staining reaction [46.5 ml of 100 mM Na_2_HPO_4_ (pH 7); 1 ml of 5% sodium azide, 2.5 ml of 1% X-GlcA, 100 mg of 5-bromo-4-chloro-3-indolyl-b-d-glucuronide dissolved in 10 ml of DMF] for 2 days at 37°C and an extraction of plant pigments in 70% EtOH at 60°C overnight, which facilitates the quantification of blue sectors using a binocular microscope.

### Preparation of pollen mother cells

The chromatin staining of the pollen mother cells was performed as described (37,38). Primary inflorescences were fixed in ethanol and acetic acid (3:1). The flower buds at different stages were washed in 0.01 M citrate buffer (pH 4.5) and digested in 0.3% cellulase (C1794, Sigma-Aldrich Chemie GmbH, Taufenkirchen, Germany) and 0.3% pectolyase (P5936, Sigma-Aldrich Chemie GmbH) in 0.01 M citrate buffer for 75 min at 37°C. Three to four flower buds each were squashed on a slide and mixed with 7 µl of 60% acetic acid. The slides were briefly incubated on a heated plate at 45°C. Finally, the reaction was finished by adding fixative [ethanol and acetic acid (3:1)], and the slides were dried using a hairdryer. The chromatin was stained with 10 µl of VECTASHIELD mounting medium with 4′6-Diamidin-2-phenylindol (DAPI) (Vector Laboratories Inc., Burlingame, California). The meiotic stages were visualized by fluorescence microscopy (Zeiss Axio Imager M1 microscope, Plan-APOCHROMAT (100x/1.4 Oil), AxioCam MR).

## RESULTS

### Defining the domain structure of AtRMI1

Previously published data for the human RMI1 homologue revealed the existence of three conserved domains (17,22,30). At the N-terminus, there is a domain of unknown function 1767 (DUF1767; pfam08585) ranging from amino acid (aa) 13 to 104 (39). Next to the DUF1767 domain, the OB-fold domain 1 (OB1) is located from aa 115 to 191. The residues 151–196 and the conserved lysine (K166) are essential for the interaction with the topoisomerase HsTOP3A and the helicase HsBLM of the human RTR complex (30). At the C-terminus of HsRMI1, a second OB-fold domain (OB2) was assigned to aa 473–625 (25,30).

**Figure 1.**
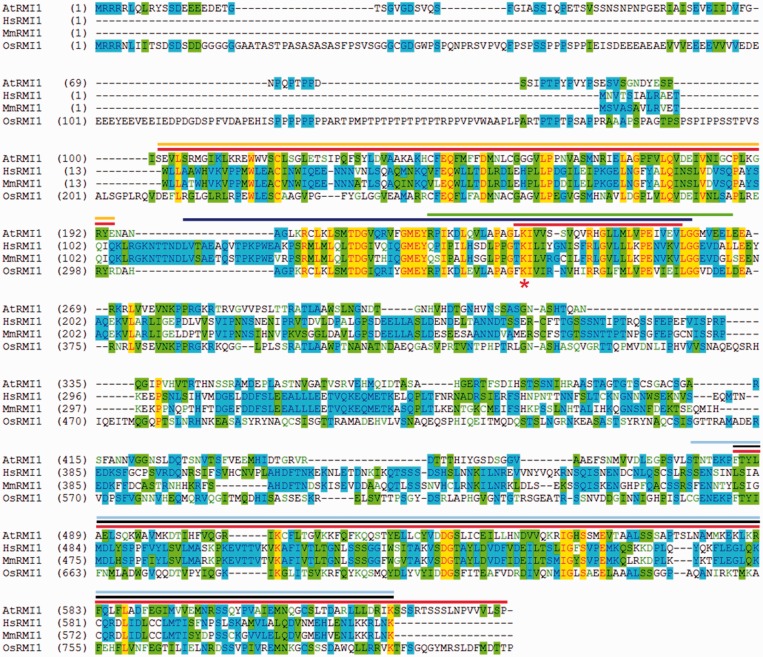
Alignment of RMI1 homologues from *A. thaliana*, *Homo sapiens*, *Mus musculus* and *Oryza sativa*. Identical amino acids for all four proteins are highlighted in yellow and for three proteins in blue. Similar amino acids are highlighted in green. The sequence regions that were deleted in the respective AtRMI1 variants are marked in red (ΔDUF: aa 101–194; ΔOB1: aa 234–258; ΔOB2: aa 485–644). The yellow overlined region in the N-terminus indicates the conserved DUF1767 domain [pfam08585; (25)]. The subsequent blue overlined sequence highlights the putative OB-fold domain 1 of HsRMI1, as described by Yin *et al.* (17). The green overlined amino acid region has been shown to be essential for the binding of HsBLM and HsTOP3A. Within the OB-fold domain 1, the conserved lysine (marked by an asterisk) has been described as an essential amino acid for interaction with HsTOP3A (30). The OB-fold domain 2 is located in the C-terminal part of RMI1 [overlined in black; (25)]. The OB-fold domain 2 of HsRMI1 is required for its interaction with HsRMI2.

The *A. thaliana RMI1* gene (At5g63540) contains an ORF of 2247 bp (AY735746) that encodes a protein of 644 aa (23). The AtRMI1 protein was shown to contain the same three domains (DUF1767, OB1 and OB2) as its human homologue (23,25). In Arabidopsis RMI1, the DUF1767 domain is located at aa 101–194. The centrally located OB1 domain ranges from aa 234 to 258. The conserved lysine K166 of HsRMI1 corresponds to K235 in AtRMI1. The conserved region of the OB-fold domain 2 contains amino acids 485–627 in *A. thaliana* (Figure 1).

### Setup of the complementation experiments

**Figure 2.**
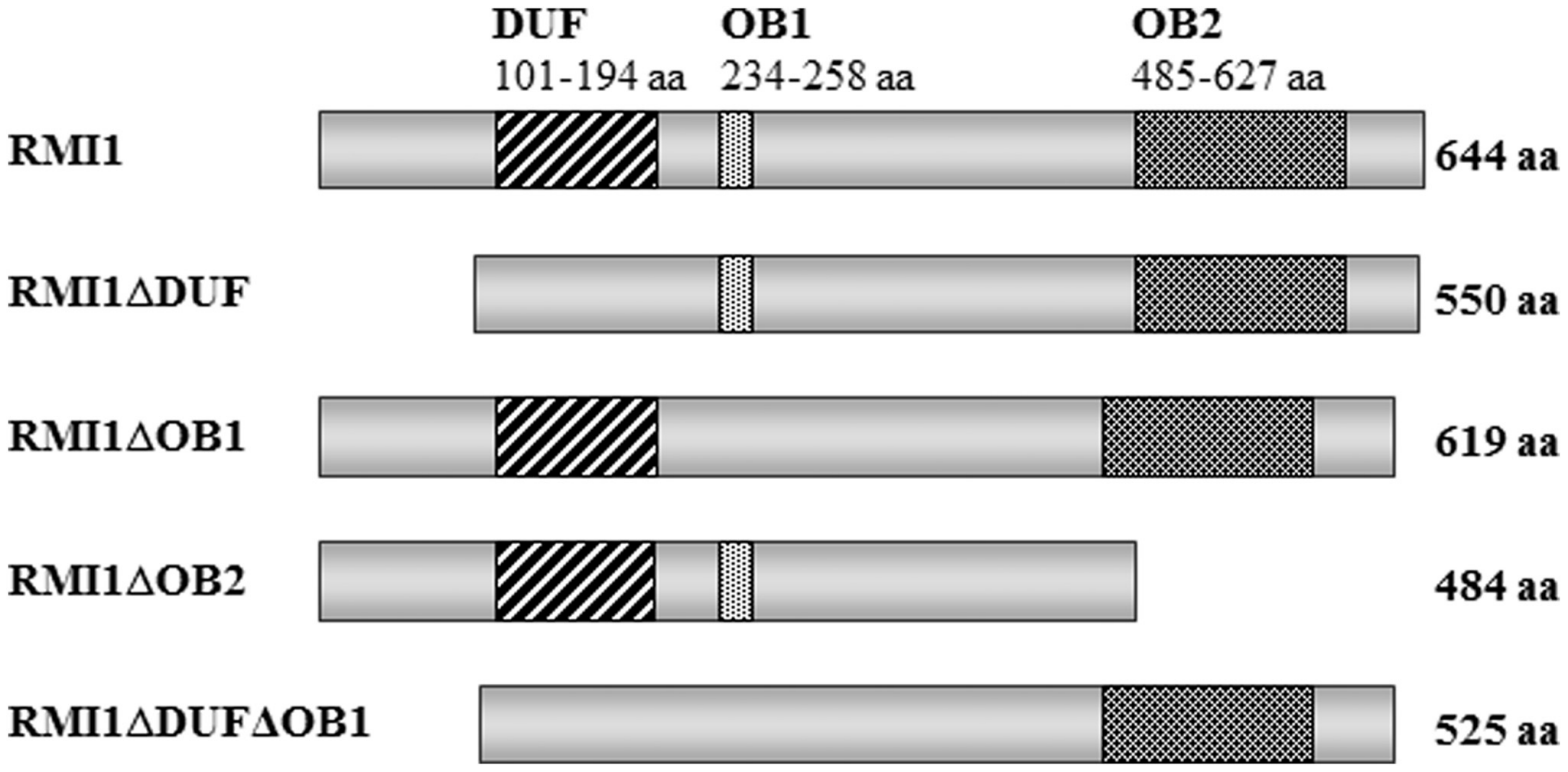
Schematic representation of the recombinant RMI1 constructs expressed in the Arabidopsis *RMI1* mutants. From top to bottom: RMI1 (644 aa), RMI1ΔDUF (550 aa), RMI1ΔOB1 (619 aa), RMI1ΔOB2 (484 aa), RMI1ΔDUFΔOB1 (525 aa). The N-terminus contains the DUF1767 domain (aa 101–194) and the OB-fold domain 1 (aa 234–258). The OB-fold domain 2 (aa 485–627) is located in the C-terminal part of the AtRMI1.

For the complementation experiments with the *Atrmi1* mutants, we cloned a full-length wild-type construct and versions of the *AtRMI1* ORF in which individual domains were deleted. The region of the DUF1767 domain that was deleted corresponds to the complete domain, ranging from aa 101 to 194. The deletion of the OB fold domain 1 contains the conserved lysine K235, but it was limited to residues 234–258. In case of the OB fold domain 2 deletion, the AtRMI1 protein was truncated after aa residue 484. The complete C-terminus from aa 484 to 644 is missing (Figure 2).

All constructs were cloned between the natural *AtRMI1* promoter and terminator to ensure their natural expression level *in planta* following transformation into wild-type, *Atrmi1-1* and *Atrmi1-2* mutant plants. The expression of the different constructs should lead to the following recombinant proteins: RMI1 (644 aa), RMI1ΔDUF (550 aa), RMI1ΔOB1 (619 aa), RMI1ΔOB2 (484 aa) and RMI1ΔDUFΔOB1 (525 aa) (Figure 2). To ensure the correct expression of the constructs, we used quantitative RT-PCR using a diagnostic amplicon present in all constructs as well as in wild-type plants. By comparing the expression of *AtRMI1* wild-type and mutant plants as high and low baselines with homozygous single locus lines, we were able to show that all lines expressed their transgene constructs more strongly than the *Atrmi1* mutant lines. Most of the transformed lines showed a higher expression of their construct than the expression of *AtRMI1* in wild-type (Supplementary Figures S1 and S2). Therefore, a lack of complementation of the mutant phenotype cannot be due to insufficient gene expression. A direct comparison of the protein concentrations could not be performed, owing to the lack of an AtRMI1-specific antibody.

For complementation we used two different *Atrmi1* mutants that we characterized previously (23). In addition to the DNA repair and somatic HR phenotypes of the *Atrmi1-2* mutant, the loss of *A. thaliana* RMI1 function is also accompanied by a meiotic recombination defect and sterile plants in the *Atrmi1-1* mutant. Thus, the *Atrmi1-1* mutant seems to be a complete knockout, whereas *Atrmi1-2* is only deficient in its somatic functions. This might be due to the genomic changes resulting from T-DNA insertions in the two lines. Although in *Atrmi1-1* the original T-DNA insertion led to a deletion of the majority of the gene spanning from the middle of exon 1 to exon 5, in *Atrmi1-2*, the T-DNA is inserted at the end of exon 5 (23). This leads to the expression of a shortened mRNA in *Atrmi1-1*, but an expression at wild-type level 5′ of the T-DNA insertion in *Atrmi1-2*. As both lines show comparable somatic phenotypes, the *Atrmi1-2* mutant, which is easier to propagate and analyse, was used for the complementation of the somatic functions, whereas the meiotic functions were addressed by the use of *Atrmi1-1.*

For each construct, four homozygous single locus lines descending from individual transformation events were tested for their sensitivity against the cross-linking agent cisplatin, the DNA methylating agent MMS and the frequency of somatic HR.

### The DUF1767 and OB1 domains are essential, and the OB2 domain is supportive for DNA repair

**Figure 3.**
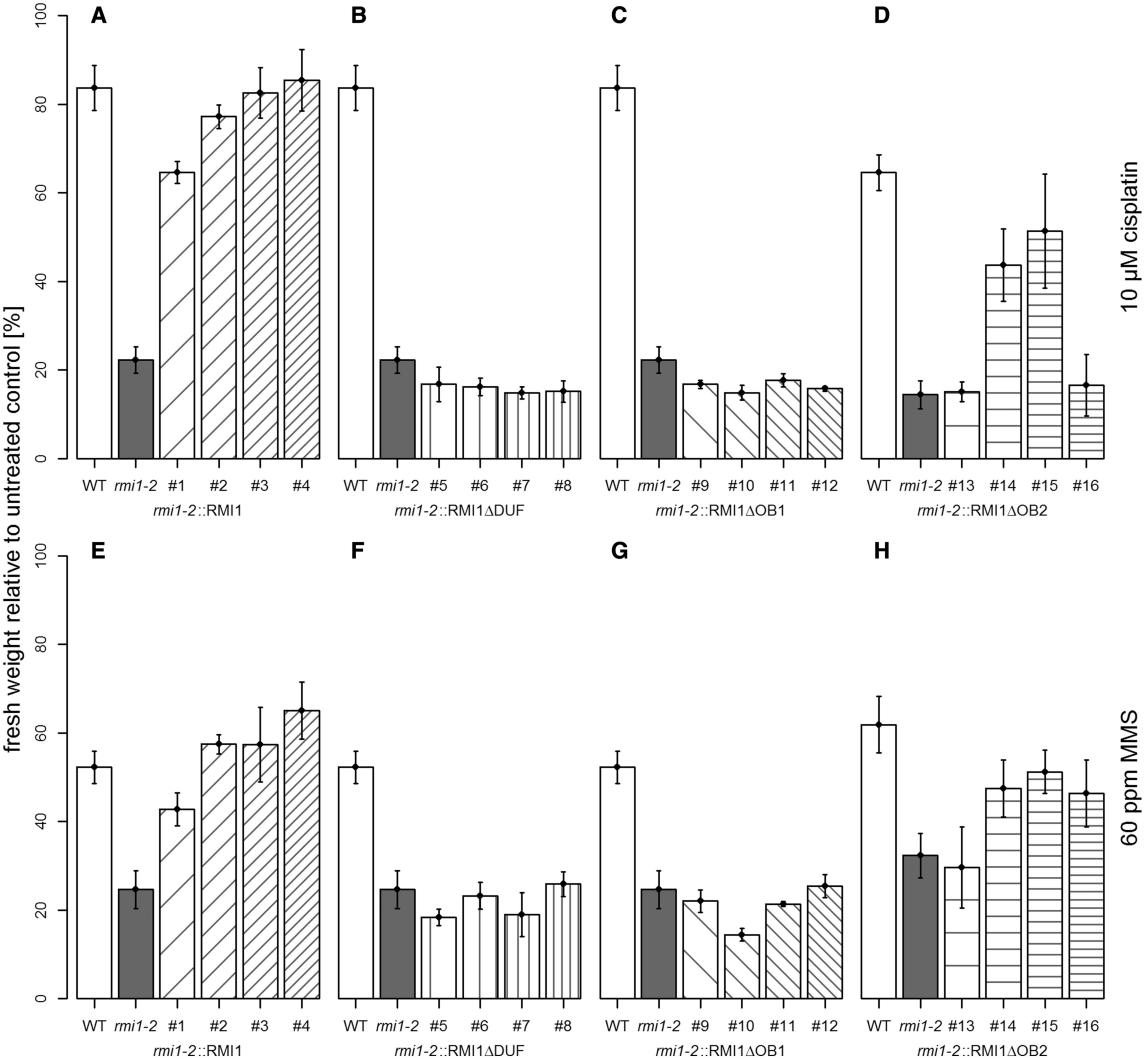
Complementation of the cisplatin and MMS sensitivity of *Atrmi1-2*. In all depicted experiments, a final cisplatin concentration of 10 µM and a final MMS concentration of 60 ppm was used. (**A**) The expression of the wild-type RMI1 construct enables the complementation of the hypersensitivity of *Atrmi1-2* against cisplatin. (**B** and **C**) The constructs RMI1ΔDUF and RMI1ΔOB1 cannot compensate for the elevated sensitivity against cisplatin. (**D**) In comparison with RMI1ΔDUF and RMI1ΔOB1, with the recombinant protein RMI1ΔOB2, some lines show complementation of the hypersensitivity against cisplatin. (**E**) Expression of wild-type RMI1 in *Atrmi1-2* rescues the hypersensitivity of the mutant against MMS. (**F** and **G**) Lines expressing the constructs RMI1ΔDUF and RMI1ΔOB1 cannot repair MMS-induced DNA damage better than the *Atrmi1-2* mutant line. (**H**) Most lines expressing a construct of RMI1 missing the OB2 domain display a repair capacity of MMS-induced DNA damage that is higher than that of the *Atrmi1-2* mutant. All experiments *n* = 3.

The enhanced sensitivity of *Atrmi1-2, Atrecq4A* and *Attop3a* against cisplatin and MMS was previously shown by Hartung *et al.* (23,24). The complementation of the hypersensitive *Atrmi1-2* mutant phenotype against cisplatin and MMS was analysed by the determination of the fresh weight of the plants challenged with either the cross-linking agent cisplatin (10 µM) or the alkylating agent MMS (60 ppm) in comparison with untreated plants. After 1 week of growing on solid medium, the plants were transferred to liquid medium and treated with cisplatin or MMS for two additional weeks. The complementation of the enhanced cisplatin and MMS sensitivity of the *Atrmi1-2* mutant was achieved by the expression of the wild-type *AtRMI1* construct (Figure 3A and E). Lines 2, 3 and 4 could fully complement the *Atrmi1-2* sensitivity against cisplatin and MMS. Line #1 showed only a partial complementation, but a significant decrease in cisplatin sensitivity (*P* < 0.001, Student’s *t*-test) and MMS sensitivity (*P* = 0.005, Student’s *t*-test) compared with the mutant line. The recombinant proteins RMI1ΔDUF and RMI1ΔOB1 could neither complement the enhanced sensitivity against cisplatin nor the enhanced sensitivity against MMS. For both constructs, the four tested lines are similar in their sensitivity to the *Atrmi1-2* mutant line (Figure 3B, C, F and G). In contrast to the constructs lacking the DUF1767 domain or the OB-fold domain 1, in lines expressing the construct RMI1ΔOB2, some could fully complement the hypersensitivity of the mutant line against both cisplatin and MMS: Lines 14 and 15 showed a sensitivity almost at wild-type level, whereas lines 13 and 16 showed hardly any complementation (Figure 3D and G).

### In contrast to the OB2 domain, the DUF1767 and OB1 domains are involved in the suppression of somatic HR

**Figure 4.**
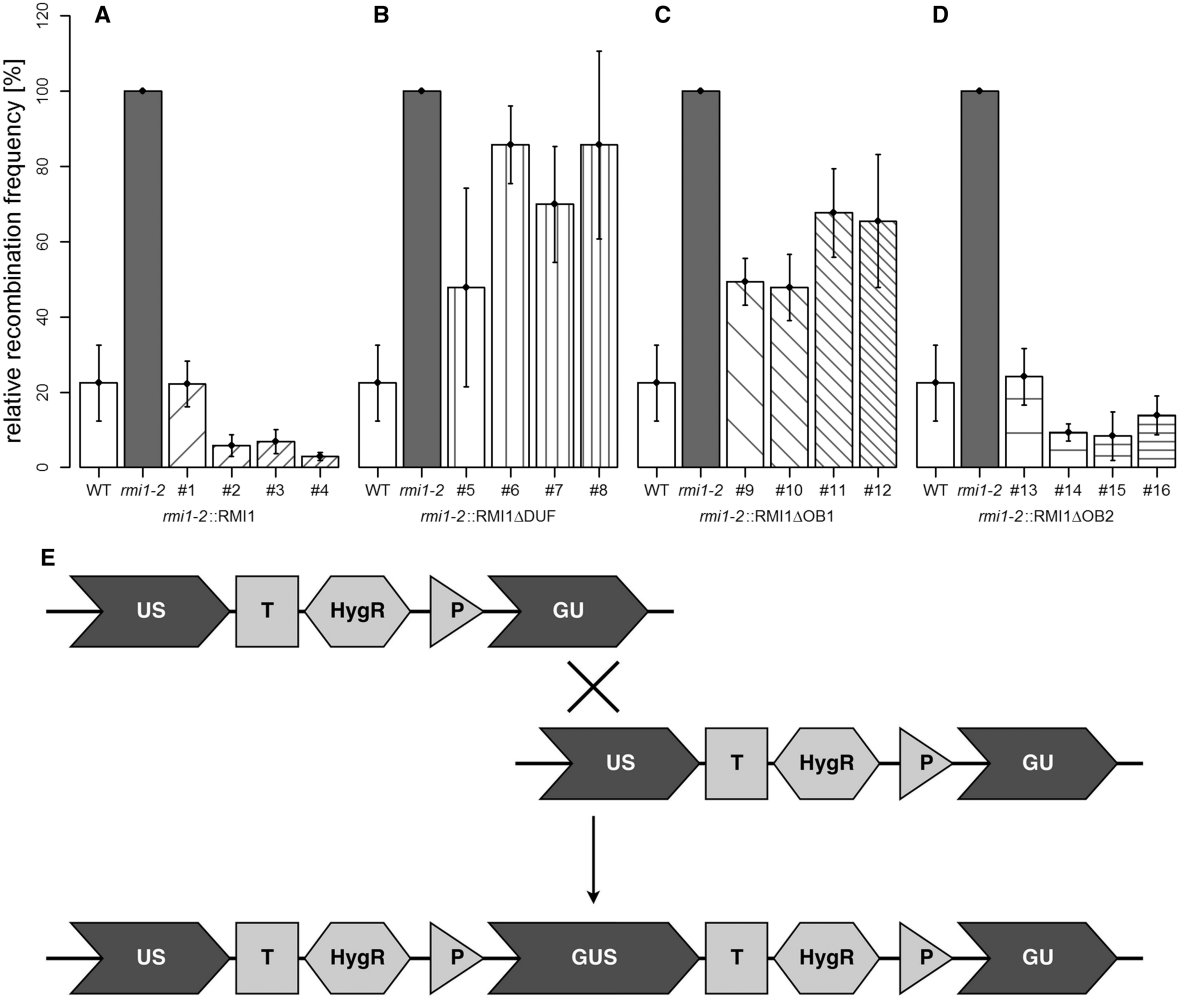
Complementation of the elevated HR frequency of *Atrmi1-2* following cisplatin treatment. HR frequency was measured in plants treated with 3 µM cisplatin. (**A**) The expression of the wild-type RMI1 construct enables the complementation of the hyper-recombination phenotype of *Atrmi1-2*. (**B** and **C**) The constructs RMI1ΔDUF and RMI1ΔOB1 cannot compensate for the enhanced frequency of recombination completely. However, the expression of these deletion constructs leads to an intermediary phenotype. (**D**) In comparison with RMI1ΔDUF and RMI1ΔOB1, the recombinant protein RMI1ΔOB2 is able to fully complement the elevated frequency of recombination. (**E**) The recombination reporter line IC9 contains two fragments of the *GUS* gene, GU and US, with homology to each other. Intermolecular HR can lead to the restoration of a fully functional *GUS* gene. Histochemical staining with X-Glc gives quantifiable blue sectors on plants indicative of HR events *in vivo*. All experiments *n* = 4.

The enhanced HR frequency of the *Atrmi1-2* mutant line was previously demonstrated by using the recombination substrate line IC9 (23). This construct harbours two non-functional fragments, ‘GU’ and ‘US’, of the *GUS* gene that share a homologous part. It is possible to restore GUS activity by recombination events between the sister chromatids or the homologous chromosomes where a full-length *GUS* gene is formed (Figure 4E). Each restoration event can be detected as a blue sector after a histochemical staining reaction with X-GlcA [(33); for a recent review, see also (40)]. The complementation of the enhanced frequency of HR of the *Atrmi1-2* mutant was measured after treatment with 3 µM cisplatin (Figure 4) and in untreated plants (Supplementary Figure S3). The plantlets were transferred into liquid medium after 1 week of growth on solid medium. After another week with or without cisplatin treatment in liquid medium, the plants were fixed and stained in a sodium azide and X-GlcA solution. In both the spontaneous and cisplatin-induced HR assays, the mutant phenotype could be successfully complemented by the expression of the wild-type RMI1 construct and the RMI1ΔOB2 construct (Figure 4A and D; Supplementary Figure S3A and D). On the other hand, the deletion of the DUF1767 domain and the OB-fold domain 1 in the N-terminal part of AtRMI1 (RMI1ΔDUF, RMI1ΔOB1) both resulted in an intermediate level of HR events compared with the HR frequency of the *Atrmi1-2* mutant and the wild-type (Figure 4B and C; Supplementary Figure S3B and C). After treatment with cisplatin, the HR frequency of all four tested lines expressing RMI1ΔOB1 is significantly different from wild-type (in all cases, *P* < 0.01, Student’s *t*-test) as well as from *rmi1-2* (#9: *P* < 0.001; #10: *P* = 0.001; #11: *P* = 0.04; #12: *P* = 0.03, Student’s *t*-test). In the lines expressing RMI1ΔDUF, only line #5 shows no significant difference from wild-type, whereas lines #6, 7 and 8 have a significantly higher HR frequency compared with wild-type (#6: *P* < 0.001; #7: *P* = 0.003; #8: *P* = 0.01, Student’s *t*-test). Furthermore, the HR frequencies of lines 5 and 7 are also significantly lower than the mutant’s (#5: *P* = 0.03; #7: *P* = 0.03, Student’s *t*-test). It is noteworthy that, as in the case of cisplatin and MMS hypersensitivity, no partial complementation of the *Atrmi1-2* mutant phenotype could be obtained by transformation with the RMI1ΔDUF and RMI1ΔOB1 constructs (see earlier in the text).

### The DUF1767 and OB1 domains have different roles in the suppression of somatic HR

**Figure 5.**
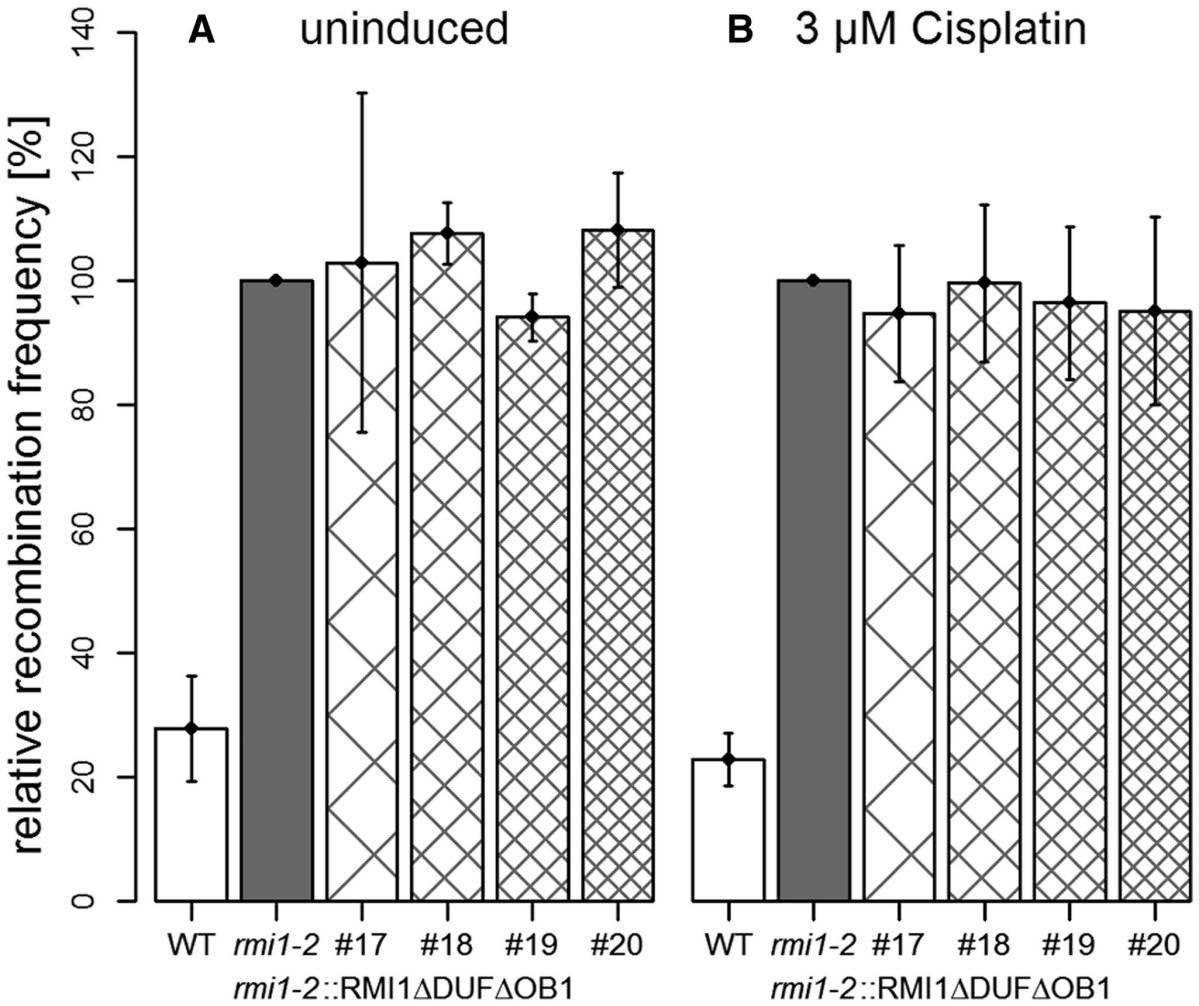
Both N-terminal domains DUF1767 and OB1 have different roles in suppressing HR. The recombination frequencies of (**A**) untreated and (**B**) cisplatin (3 µM) treated plants. In contrast to the RMI1ΔDUF and RMI1ΔOB1 constructs, the expression of the recombinant RMI1ΔDUFΔOB1 construct does not lead to any type of complementation of the hyper-recombination phenotype of the *Atrmi1-2* mutant. All experiments *n* = 3.

The previous results indicated that both N-terminal domains DUF1767 and OB1 have some function in the suppression of somatic HR. However, both could be involved in the same type of reaction that would require AtRMI1 for some but not all forms of HR suppression detected by our assay system. On the other hand, both domains might contribute, at least partially, in an independent way to the suppression phenotype so that the loss of both domains would lead to a complete loss of suppression. To discriminate between these possibilities, we generated a construct missing both the DUF1767 domain and the OB-fold domain 1 (Figure 2). After transformation of the *Atrmi1-2* mutant, four different independent transgenic lines were isolated and analysed. The expression of the recombinant protein RMI1ΔDUFΔOB1 did not significantly change the frequency of HR of the *Atrmi1-2* mutant either with or without cisplatin treatment (Figure 5). On the other hand, the HR frequency of all lines was significantly different from that of the wild-type without (#17: *P* = 0.03; #18: *P* = 0.02; #19: *P* = 0.003; #20: *P* < 0.001, Student’s *t*-test) or with cisplatin treatment (#17: *P* = 0.001; #18: *P* = 0.003; #19: *P* = 0.004; #20: *P* = 0.01, Student’s *t*-test). The fact that *Atrmi1* plants expressing an AtRMI1 protein without both the DUF1767 and OB domain 1 show a recombination frequency that is higher than that of plants expressing an AtRMI1 where only one of the domains is missing (compare Figure 5 with Figure 3B and C; Supplementary Figure S3B and C) indicates that both domains have at least partially non-overlapping functions in the suppression of somatic HR.

### DUF1767 and OB1 but not OB2 are essential for meiotic recombination

**Figure 6.**
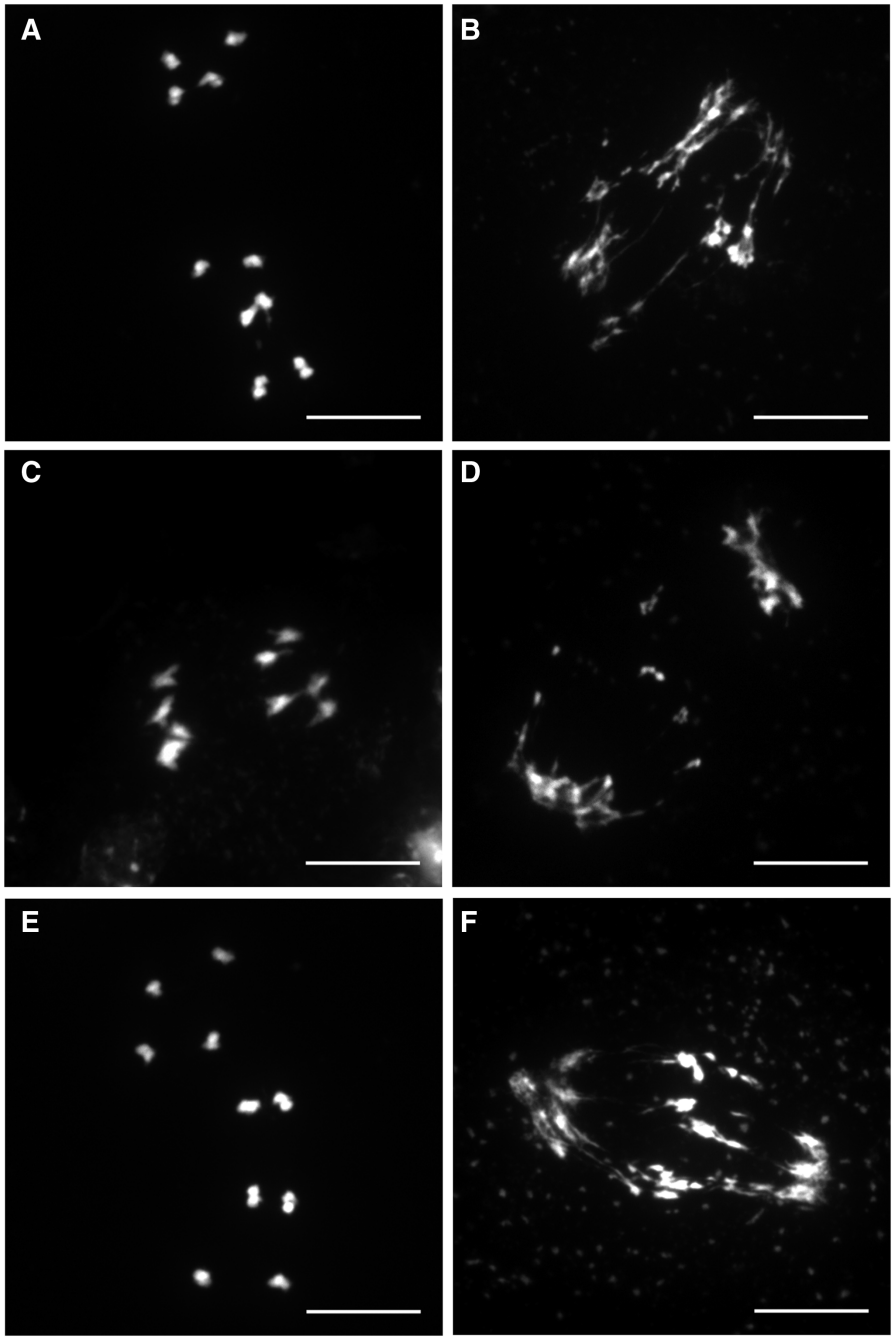
Rescue of meiotic defects in *Atrmi1-1*. In the wild-type anaphase I of meiosis, homologous chromosomes are separated to the poles. In the anaphase I of *Atrmi1-1*, however, unresolved recombination intermediates still connect homologous chromosomes, leading to the formation of chromatin bridges. The expression of the proteins RMI1 and RMI1ΔOB2 restores homologous chromosome recombination and enables the separation of the homologous chromosomes without chromosome fragmentation. The expression of the proteins RMI1ΔDUF and RMI1ΔOB1 does not change the *Atrmi1-1* chromatin fragmentation phenotype. (**A**) wild-type, (**B**) *Atrmi1-1*, (**C**) *Atrmi1-1*::RMI1, (**D**) *Atrmi1-1*::RMI1ΔDUF, (**E**) *Atrmi1-1*::RMI1ΔOB2, (**F**) *Atrmi1-1*::RMI1ΔOB1. Bars = 10 µm.

In addition to the DNA repair and somatic HR phenotypes, the complete loss of *A. thaliana* RMI1 is also accompanied by a meiotic recombination defect leading to sterile plants. Therefore, the lines expressing the recombinant proteins RMI1, RMI1ΔDUF, RMI1ΔOB1, RMI1ΔOB2 and RMI1ΔDUFΔOB1 were tested for their ability to complement the sterile phenotype and the meiotic arrest of the *Atrmi1-1* mutant line. The expression of the different constructs was measured by quantitative RT-PCR (Supplementary Figure S2). To define the role of the individual domains in meiotic recombination, we analysed the number of fertile *Atrmi1-1* T1 plants after the transformation of hemizygous *Atrmi1-1* plants with the AtRMI1 full-length construct and the four deletion constructs (RMI1, RMI1ΔDUF, RMI1ΔOB1, RMI1ΔOB2 and RMI1ΔDUFΔOB1). Transformed plants were selected by PPT resistance mediated by the inserted T-DNA, and the *AtRMI1* mutation was genotyped by PCR. The fertility of homozygous *Atrmi1-1* plants was checked by counting the number of seed-bearing T1 plants in the greenhouse (Table 1). Furthermore, we quantified the fertility by assaying the mean number of seeds per silique after the transformation with the five different complementation constructs in the T1 generation. Only the expression of the recombinant proteins RMI1 and RMI1ΔOB2 enabled the development of fertile plants, and a fertility level comparable with that of wild-type plants was also observed with the RMI1 complementation constructs, whereas plant lines expressing RMI1ΔDUF and RMI1ΔOB1 were sterile (Table 1, Supplementary Figure S4). The remarkable meiotic phenotype of the *Atrmi1-1* mutant becomes apparent in anaphase I of the first meiotic division. In comparison with anaphase I in the pollen mother cells of wild-type plants (Figure 6A), the chromosomes in the meiotic cells of *Atrmi1-1* cannot be separated properly, and dramatic chromosome fragmentation can be observed (Figure 6B), which leads to an arrest at the end of meiosis I. Therefore, the stages of the second meiotic division cannot be detected in *Atrmi1-1* (23,25). Following the expression of the different recombinant AtRMI1 variants, we could detect a normal progression of meiosis only in the lines expressing the wild-type construct and the RMI1ΔOB2 construct (Figure 6C and E). These constructs allowed a complete passage of the two meiotic divisions with anaphase I stages resembling wild-type plants and a characteristic microspore tetrad indicating the completion of meiosis II. Contrary, the recombinant proteins RMI1ΔDUF and RMI1ΔOB1 could not alter the meiotic phenotype of the *Atrmi1-1* mutant (Figure 6D and F). In these lines, only defective anaphase I stages were observed, and no stages of the second meiotic division were detectable. The complementation with the RMI1ΔOB2 construct was as efficient as using the complete ORF, which demonstrates that the OB-fold domain 2 is completely dispensable for the meiotic functions of AtRMI1.

**Table 1.** Complementation of *Atrmi1-1* sterility by the expression of recombinant RMI1 proteins

	Plants tested	Fertile plants	Fertile plants (%)
*Atrmi1-1*	58	0	0
*Atrmi1-1*::RMI1	62	61	98
*Atrmi1-1*::RMI1ΔDUF	52	0	0
*Atrmi1-1*::RMI1ΔOB1	54	0	0
*Atrmi1-1*::RMI1ΔOB2	52	51	98
*Atrmi1-1*::RMI1ΔDUFΔOB1	49	0	0

Plant lines expressing full-length RMI1, RMI1ΔDUF, RMI1ΔOB1, RMI1ΔOB2 and RMI1ΔDUFΔOB1 in an *Atrmi1-1* mutant background were tested for their ability to form progeny by counting the number of seed-bearing T1 plants. The sterile phenotype of the mutant could only be rescued by the wild-type RMI1 construct and the RMI1ΔOB2 construct.

## DISCUSSION

The evolutionarily highly conserved RTR complex, consisting of a RecQ helicase, a homologue of the type IA topoisomerase 3 or 3α and the structural protein RMI1, catalyses the dissolution reaction of HR intermediates. Thus, it suppresses the formation of CO products and contributes significantly to the maintenance of genome stability (41,42). RMI1 plays an essential role in the RTR complex, which has been demonstrated for *RMI1* mutants of different organisms. The characterization of *rmi1* mutants of *A. thaliana* revealed that AtRMI1 has an important function in the processing of replication-associated DNA damage indicated by the reduced efficiency of repairing DNA damage induced by cisplatin and MMS and the suppression of somatic HR (23). Surprisingly, AtRMI1 also has an essential role in the progression of meiosis (23,25), a phenomenon not previously reported for other RMI1 homologues. In the present report, we were now able to define the requirement of the different domains of AtRMI1 for these different functions.

Notably, although AtTOP3A has a similar role in meiosis (23), for the BLM homologue AtRECQ4A this is clearly not the case (27). Therefore, it seems that during meiosis in plants, a complex different to the classical RTR complex is involved in the processing of recombination intermediates. Until now, it has been unclear whether a DNA helicase is involved in this process and, if so, which one. All of our efforts to identify a DNA helicase with a similar meiotic phenotype in Arabidopsis have failed until now (42). In light of this peculiarity, it is of special interest to elucidate the domain requirements for the different somatic and meiotic functions of AtRMI1. Although the protein possesses no catalytic function, the comparison of the roles of the individual domains for different functions should give us an idea of the complexity of the AtRMI1 interactions. Previous studies on the role of the conserved domains DUF1767, OB-fold domain 1 and OB-fold domain 2 were performed with HsRMI1 *in vitro* (30,43).

Our article presents the first functional domain analysis of AtRMI1 *in vivo.* Previously, the role of different domains in RMI1 homologues was only defined *in vitro* or in frame of an interaction analysis (22,30). Similar to the mammalian homologue, three domains can be identified in the AtRMI1 protein. The DUF1767 domain in the N-terminal part of the protein was proposed to be important for the interaction with other complex partners of the RTR complex and, to a minor degree, to be essential for the proper folding of the RMI1 protein and the stability of the entire complex (28,29). Furthermore, although no biochemical function is known for DUF1767 domains (pfam08585), they were identified by bioinformatics to be located in a number of proteins on the N-terminal side of ubiquitin-binding and nucleic acid-binding domains. In the case of RMI1, the DUF1767 is on the N-terminal side of the OB-fold domain 1, which is required for the interaction of RMI1 with the type IA topoisomerase and the RecQ helicase (30). The third conserved domain of AtRMI1 is the OB-fold domain 2 in the C-terminal part of the protein. In humans, the OB-fold domain 2 has been identified as the domain of HsRMI1 that interacts with another member of the RTR complex, *Hs*RMI2 (which contains the OB-fold domain 3) (21,44). The interaction of HsRMI1 and HsRMI2 has been shown to enhance the stability of the RTR complex and to stimulate the dissolution reaction *in vitro* (22).

One major approach of this work was to express AtRMI1 protein variants with deleted domains in *Atrmi1* mutant plants. As even small changes in amino acid sequences can influence folding and hence structure and activity of proteins, one has to be careful in the interpretation of data acquired with such constructs. We were not able to directly measure the expressed proteins *in planta*, but we are confident that the kind of intermediate effects on HR frequency seen in our experiments are not likely due to a general destabilization of the proteins involved. Furthermore, similar experiments have been performed with RMI1 and other proteins of the RTR complex *in vitro* and *in vivo*, showing stable expression of protein variants (22,30,45). Additionally, the structures of the two *Atrmi1* mutant lines used in this study have to be considered. Line *Atrmi1-2*, which was used for somatic analyses, is able to form a transcript of the gene 5′ of the inserted T-DNA (23). This fragment might also enable the cell to form a protein fragment of the N-terminal part of AtRMI1 containing the DUF1767 and OB1 domains. However, the expression of a construct missing the OB2 domain (RMI1ΔOB2), which should essentially form a protein similar to the hypothetical protein expressed in the mutant line *Atrmi1-2*, can complement some phenotypes of this mutant. Together with the observation that the full knockout line *Atrmi1-1* displays a comparable somatic phenotype to *Atrmi1-2*, it is highly likely that there is only some minor activity left in *Atrmi1-2*. Furthermore, the fact that we were able to complement the *Atrmi1-2* DNA repair and HR phenotypes to wild-type levels can be taken as a strong indication that the mutant phenotype is not due to the expression of a negatively complementing protein fragment.

The results of our *in vivo* domain analysis indicate that AtRMI1 is involved, at least partially, in a number of different steps of DNA cross-link repair, suppression of somatic HR and meiotic recombination. The DUF1767 domain and the OB-fold domain 1 are both essential for DNA cross-link repair. Moreover, the OB-fold domain 2 also plays some role in this pathway. Owing to random DNA integration following transformation of *A. thaliana*, rarely all transformed lines are phenotypically similar to each other. In the case of the lines expressing construct RMI1ΔOB2, two of the four lines tested were not different from the mutant, whereas the other two were able to render the plants more resistant to cisplatin and MMS treatment. As some transgenic lines are expected to be located in transcriptionally silent loci, we tested the expression of the RMI1 construct in all four lines. However, expression levels did not correlate with cisplatin or MMS sensitivity levels. Therefore, differing transcriptional activities cannot be the reason behind the differences. As specific insertion loci were not determined for each line, there might be unknown kinds of locus-specific indirect effects. Nevertheless, at least half of the tested lines were able to repair cisplatin or MMS-induced DNA damage almost as efficiently as wild-type.

Thus, all three domains are involved in this type of repair reaction. This contrasts with the behaviour in somatic and meiotic recombination. The OB-fold domain 2 is dispensable for both types of recombination reactions. Nevertheless, our results indicate that the role of AtRMI1 in both pathways is not identical. Although individually the DUF1767 as well as OB-fold domain 1 are absolutely essential for the progression of meiosis, a partial suppression of somatic recombination can occur if one or the other domain is not functional. This shows that the AtRMI1 DUF1767 domain is not simply an N-terminal extension of the first OB-fold domain, but that it must possess a function distinct from the OB1 domain.

AtRMI1 is involved in different types of reactions in DNA repair and HR. During these reactions, it interacts to different extents with other proteins via its respective domains. Our analysis shows that the OB-fold domain 2 from AtRMI1 is dispensable for HR in somatic and meiotic cells. We found that it is possible to fully complement both the elevated frequency of HR in somatic cells and the meiotic defect of the *Atrmi1* mutants with a recombinant protein that does not contain the OB-fold domain 2. According to these results, the interaction of AtRMI1 with a putative AtRMI2 is dispensable for recombination functions. HsRMI2 could be identified as a fourth essential component of the RTR complex in humans but not in yeast (21,22). An RMI2 homologue is also present in *A. thaliana*, which possesses a similar predicted structural organization needed for the interaction of HsRMI2 and HsRMI1 (29,31). As the OB-fold domain 2 of AtRMI1 is required to a certain extent for the repair of DNA damage induced by methylating or cross-linking agents, it might well be that such a complex exists *in planta* and is involved in certain pathways of DNA repair. Therefore, it will be an interesting task to define the biological role of AtRMI2 in detail.

The fact that we detected partial complementation in some assays can be taken as a hint that there might be more than just one pathway addressed with this assay. This applies for the role of OB-fold domain 2 in DNA repair as well as the DUF1767 and the OB-fold domain 1 in the suppression of somatic HR. All this indicates that RMI1 has a much more complex role in mediating DNA processing enzymes than originally anticipated. In addition to dissolving dHJs, the RTR complex is involved in other steps of HR, such as the resection of double-stranded DNA ends in yeast. Interestingly, no active topoisomerase is required for this function (46). It has also been demonstrated in Drosophila that the respective BLM homologue is involved in the D-loop formation step of the synthesis-dependent strand-annealing pathway of HR (47). Indeed, a defect in synthesis-dependent strand-annealing was also reported for the respective AtRECQ4A mutant (48). It is not clear whether the other partners of the RTR complex are also involved in this reaction. It was previously reported that Rmi1 in yeast also contributes to sister chromatid cohesion; however, it is not clear whether all other partners of the RTR complex are also required for this reaction (49,50). In addition to its interactions within the RTR complex, it has been demonstrated that RMI1 interacts with the DNA translocase FANCM in mammals (44,51). It will therefore be interesting to define its interactions with the respective Arabidopsis homologue (38,52).

## SUPPLEMENTARY DATA

Supplementary Data are available at NAR Online.

## FUNDING

German Research Foundation DFG [Pu 137/11] and the European Research Council ERC [ERC-2010-AdG_20100317 COMREC]. Funding for open access charge: German Research Foundation [Pu 137/11].

*Conflict of interest statement*. None declared.

## Supplementary Material

Supplementary Data
