# Supplementary material for: Different functions for the domains of the Arabidopsis thaliana RMI1 protein in DNA cross-link repair, somatic and meiotic recombination
Source: Nucleic Acids Res. 2013 Aug 16;41(20):9349–60. doi: 10.1093/nar/gkt730 (PMC3814364; doi:10.1093/nar/gkt730)
Supplement: Supplementary Data [file supp_41_20_9349__index.html]

Different functions for the domains of the Arabidopsis thaliana RMI1 protein in DNA cross-link repair, somatic and meiotic recombination — Different functions for the domains of the Arabidopsis thaliana RMI1 protein in DNA cross-link repair, somatic and meiotic recombination — Supplementary Data 

# Different functions for the domains of the *Arabidopsis thaliana* RMI1 protein in DNA cross-link repair, somatic and meiotic recombination

## Supplementary Data

files

**Files in this Data Supplement:**

- Supplementary Data - pdf file
