# Supplementary material for: Different functions for the domains of the Arabidopsis thaliana RMI1 protein in DNA cross-link repair, somatic and meiotic recombination
Source: Nucleic Acids Res. 2013 Aug 16;41(20):9349–60. doi: 10.1093/nar/gkt730 (PMC3814364; doi:10.1093/nar/gkt730)
Supplement: Supplementary Data [file supp_gkt730_nar-01538-d-2013-File008.pdf]

Supplementary Figure 1

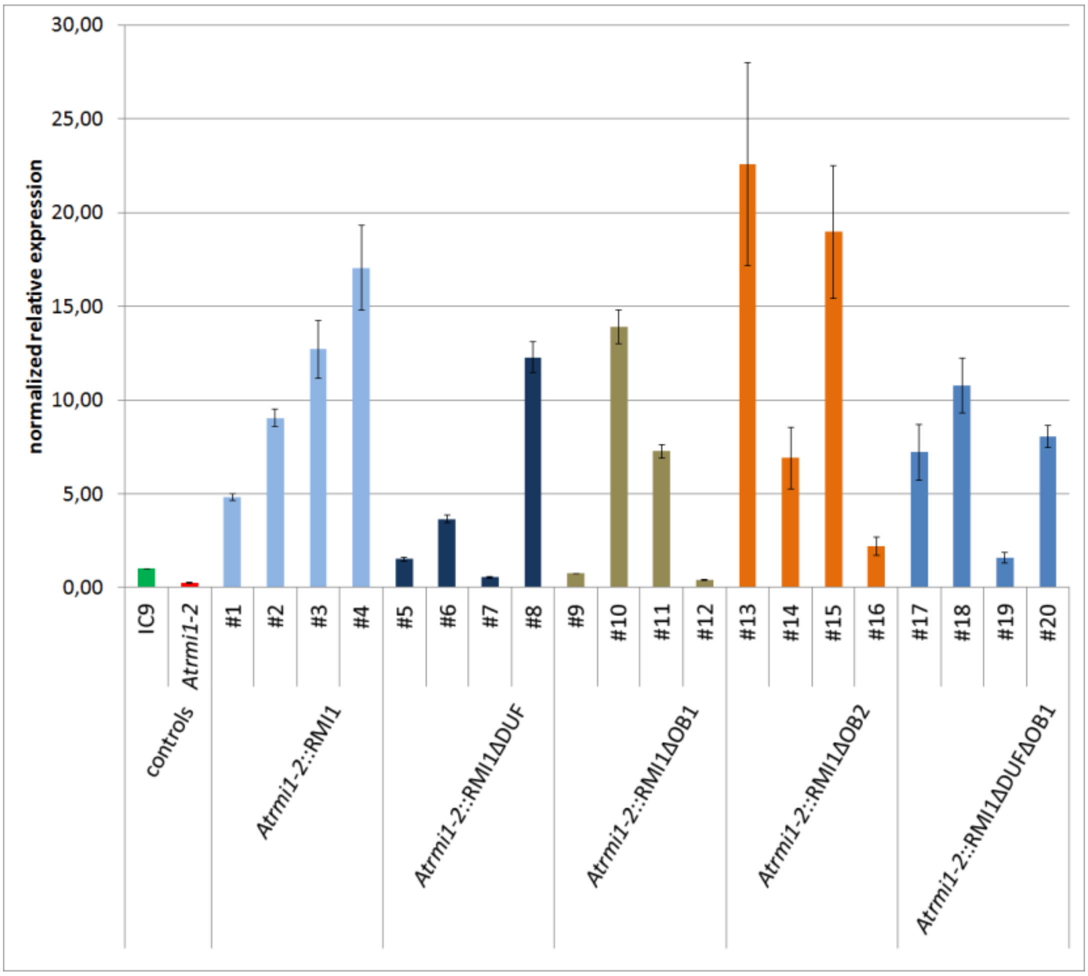

Supplementary Figure 1: Quantitative RT-PCR data of the expression of 5 constructs (RMI1, RMI1ΔDUF, RMI1ΔOB1, RMI1ΔOB2, RMI1ΔDUFΔOB1) in the homozygous single-locus lines of *Atrmi1-2*. We measured the expression of a diagnostic amplicon of *AtRMI1* present in all tested lines relative to the expression in wild-type plants. *Atrmi1-2* mutant plants show a strongly reduced *AtRMI1* expression. All transformed lines display higher expression of the respective construct than the mutant, and most lines were even higher than the wild-type line IC9. Line IC9 is a descendant of wild-type Col-0 containing a HR reporter construct (see Figure 4E; Molinier et al. (2004) Interchromatid and interhomolog recombination in Arabidopsis thaliana. Plant Cell, 16, 342-352). All experiments n = 3.

Supplementary Figure 2

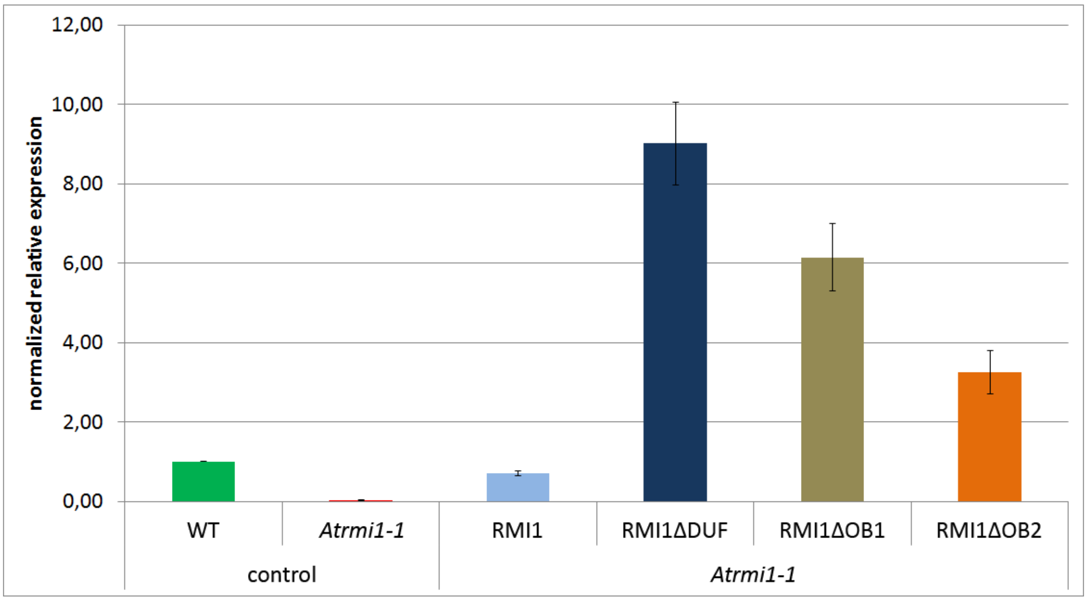

Supplementary Figure 2: Quantitative RT-PCR data of the expression of 4 constructs (RMI1, RMI1ΔDUF, RMI1ΔOB1, RMI1ΔOB2, RMI1ΔDUFΔOB1) in the homozygous single-locus lines of *Atrmi1-1*. We measured the expression of a diagnostic amplicon of *AtRMI1* present in all tested lines relative to the expression in wild-type plants. *Atrmi1-1* mutant plants show a strongly reduced *AtRMI1* expression. All transformed lines display a higher expression of the respective construct than the mutant, and most lines were even higher than the wild-type. All experiments n = 3.

Supplementary Figure 3

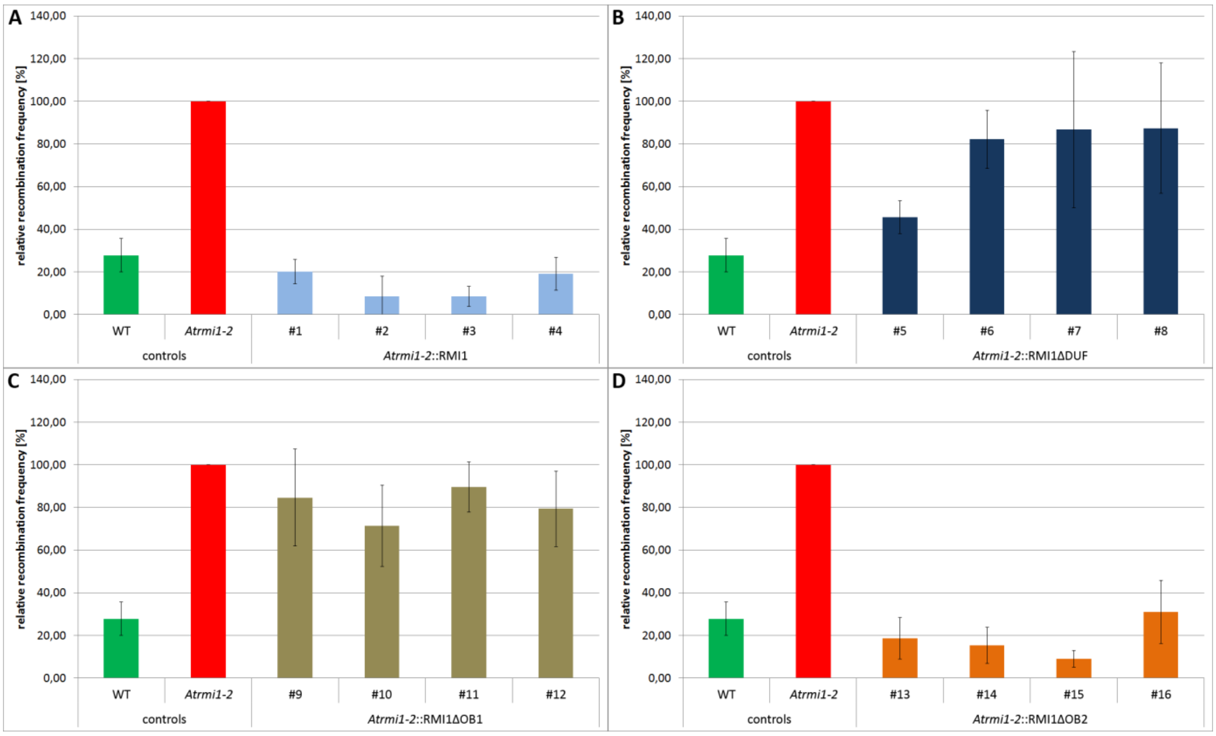

Supplementary Figure 3: Complementation of the elevated HR frequency of the *Atrmi1-2* mutant (untreated). (A) The expression of the wild-type RMI1 construct enables the complementation of the hyper-recombinative phenotype of *Atrmi1-2*. (B, C) The constructs RMI1ΔDUF and RMI1ΔOB1 cannot compensate for the enhanced frequency of recombination completely. The expression of these deletion constructs leads to an intermediary phenotype. (D) In comparison, the recombinant protein RMI1ΔOB2 shows no complementation of the elevated frequency of recombination. All experiments n = 3.

Supplementary Figure 4

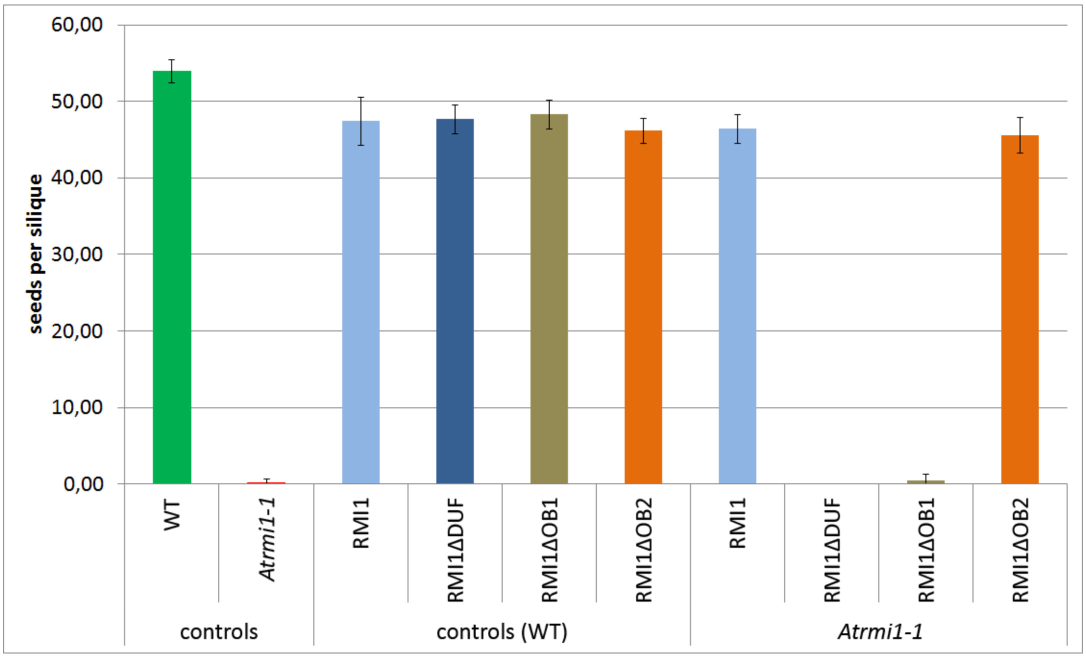

Supplementary Figure 4: Fertility tests of transformed *Atrmi1-1* mutant lines. The number of seeds per silique was assessed as a measure for fertility. For each homozygous single-locus line expressing the recombinant proteins RMI1, RMI1ΔDUF, RMI1ΔOB1, RMI1ΔOB2, 5 plants (5 siliques each) were analysed. A comparable amount of seeds per silique could only be achieved by the expression of the wild-type RMI1 construct and the RMI1ΔOB2 construct.
